# Perceived Environmental Factors Associated with Obesity in Libyan Men and Women

**DOI:** 10.3390/ijerph15020301

**Published:** 2018-02-09

**Authors:** Hamdi Lemamsha, Chris Papadopoulos, Gurch Randhawa

**Affiliations:** 1 Faculty of Medical Sciences, University of Omar Al-Mukhtar, Al-Bayda Campus, Labraq Road, Al-Bayda B1L12, Libya; dr_hamdi_lemamsha@outlook.com; 2 Institute for Health Research, University of Bedfordshire, Putteridge Bury Campus, Hitchin Road, Luton LU2 8LE, UK; chris.papadopoulos@beds.ac.uk

**Keywords:** obesity, Libya, neighbourhood environment

## Abstract

*Background*: There is a lack of research pertaining to the links between built environment attributes and obesity in adults in the Eastern Mediterranean Region. In the Libyan context, no previous studies have been conducted to investigate this relationship. Therefore, the aim of this study was to examine associations between perceived neighbourhood built environmental attributes and obesity among Libyan men and women. The prevalence of overweight and obesity was also assessed. *Methods:* A cross-sectional study design was used for the population-based survey in Benghazi, Libya. A multi-stage cluster sampling technique was used to select Libyan adults from the Benghazi electoral register. The Physical Activity Neighbourhood Environment Scale (PANES) was used to measure participants’ perception of neighbourhood environmental factors. Using the Tanita BC-601 Segmental Body Composition Monitor and a portable stadiometer, anthropometric measurements were taken at a mutually agreeable place by qualified nurses. *Results*: Four hundred and one Libyan adults were recruited (78% response rate). Participants were aged 20–65 years, 63% were female, and all had lived in Benghazi for over 10 years. The prevalence of obesity and overweight was 42.4% and 32.9% respectively. A significant association was found between BMI and 6 neighbourhood environment attributes, specifically: street connectivity, unsafe environment and committing crimes at night, and neighbourhood aesthetics. For men only, these were: access to public transport, access to recreational facilities, and unsafe environment and committing crimes during the day. The attribute ‘residential density zones’ was only significant for women. *Conclusions*: The study suggests that Libyan people are at risk of living in neighbourhoods with unsupportive environmental features of physical activity, which are likely to promote obesity of both genders. The findings of this study could inform Libyan health policies about interventions in the obesogenic environments that might slow the obesity epidemic and contain the public health crisis. This study suggests that further research is needed, within the Libyan context, to explore the impact of the neighbourhood environment attributes on contributing to increased obesity.

## 1. Background

Obesity is understood to be an energy imbalance between calories consumed and calories expended which results in abnormal or excess fat accumulation in adipose tissue, typically at least 30% above the recommended body weight, and leading to health impairment [1,2]. Overweight is defined as having a body mass index (BMI is used as a screening tool and is defined as weight in kilograms divided by height in metres squared) between 25.0 and 29.9 kg/m^2^, while obesity is a BMI a BMI equal to 30 kg/m^2^ or more [1,2]. Obesity is a complex, bio-psychosocial medical condition with a multifaceted aetiology [1,2,3]. Obesity experts now concur that a global obesity epidemic exists—termed “globesity”—which is a major international public health crisis [4,5]. To understand the reasons behind a dramatic increase in obesity epidemic globally, it is necessary to investigate causes not only at the individual (intrapersonal) level—comprising changes in genes, biology, and psychology—but also at the level of the local environment, including the urban built environment [6]. Public health researchers and obesity experts have blamed city-planners for creating ‘obesogenic environments’ through land-use patterns and transport systems planners which encourage people to eat unhealthily and not exercise enough, which is potentially fuelling the obesity epidemic [7,8,9]. 

To assess what aspects of obesogenic environments support being obese, the following elements should be considered: (1) Accessibility—distance to shops and work; distance to a green space or park; cost of healthy food; cost of a physical-activity facility; (2) Availability—types of food outlets available in the local area; the quality of green space; and the quality of food in the local area and; (3) Perceptions—perceptions of safety in parks and perceptions of food provided in food outlets and the value of healthy foods [8,10,11]. Further prominent neighbourhood attributes include a safe and unsafe environment, household vehicle ownership [6,12,13] and ‘neighbourhood walkability’, often categorised according to the ‘3Ds,’ i.e., population Density, land-use Diversity, and pedestrian-friendly streets Design [14,15,16,17].

The first ‘D’—population Density—is debated first. Numerous epidemiological studies have shown that higher residential density is associated with significantly lower BMIs [18,19,20,21,22]. In contrast, other studies suggest that urban sprawl often generates low-density residential areas, which is associated with an increased risk of obesity in both genders [23,24,25,26]. However, other studies have failed to find a significant association between residential density and BMI, in either gender [27,28]. The second ‘D’—land-use Diversity—is a common attribute of the built environment that encourages neighbourhood walkability. Numerous of studies have shown a reverse association between land-use mix and body-weight: the greater the land-use mix, the lower the BMIs of both genders [12,27,29,30,31,32]. However, other studies have found a positive association between the two: the greater land-use mix, the higher the BMIs of both genders [33]. The third ‘D’—pedestrian-friendly streets Design—is a feature of neighbourhood walkability referring to pedestrian- and transit-friendly street design and streetscape design [17,34]. A number of epidemiological studies have shown mixed and inconsistent results regarding the association between factors belonging to pedestrian- and transit-friendly street design and obesity of both genders [35,36,37], whether the neighbourhood attributes were assessed using objective indicators or respondents’ perceptions [11]. Nevertheless, there is a consensus among researchers that a better-designed neighbourhood tends to be associated with a lower likelihood in BMI of both genders [38]. Studies consistently suggest that increased access to and use of public transit are negatively correlated with BMI, while those who use private transport have higher BMI of both genders [22,39,40]. Similarly, a recent study showed that switching from private motor vehicle to public transport or active travel was associated with a decline in BMI of both genders [41]. Such disparities in the associations between the ‘3Ds’ and body-weight outcomes, make it difficult to draw inference about such relationship in both developed and developing countries. 

Despite most studies to date that examine associations between built environments, physical activity and obesity being conducted in developed countries [6,14,28,42], there is a dearth such studies in developing countries, including China [43,44,45], South Africa [46], Iran [47], and Nigeria [48]. There is, however, a distinct absence of such studies in the Arab region. Despite the significance of neighbourhood environment factors in obesity research, there is still a significant knowledge gap about the association between built environments and obesity in developing countries within the Eastern Mediterranean Region (EMR) [49]. Therefore, this study aimed to examine associations between perceived neighbourhoods built environmental attributes and obesity among Libyans. The prevalence of overweight and obesity was also assessed. To our knowledge, this is the first study not only in Libya but also in the EMR, and the second study in African (the first was in Nigeria conducted by Oyeyemi et al. (2012) [49]. Hence, it is crucial to address the built-environment factors, which Libyans are likely to be exposed to, over the course of their lives as predictor variables, which may contribute to, or protect, against the obesity epidemic.

## 2. Methods

The electoral register for Benghazi Council was used as the sampling frame for this study. The study utilised a multi-stage cluster sampling technique in three stages to draw a total sample of 512 participants, using Benghazi’s 11 parliamentary constituencies [50,51] as the clusters. Listed alphabetically they are Al-Break, Al-Keisha, Al-Sabre, Al-Salami-El-Garb, Al-Salmani-ElSharki, Al-Uruba, Benghazi al-Jadida, Bu Atni, Benina, Garyounis, and Madinat Benghazi. Stage One comprised a random sampling of parliamentary constituencies; Stage Two involved sampling the polling districts, whilst Stage Three entailed the random sampling of individuals. 

Since the actual sample size of my study was computed and estimated to be 384 Libyan adults (Sample Size (SS) = $\frac{1.96^{2}\left(0.5\right)\times \left(1-0.5\right)}{0.05^{2}}$ =384 individuals) and the assumption of the response rate that was proposed to be 75%, thus, the final calculated sample size for this study was 512 Libyan adults (384/0.75 = 512).

In the first phase, Primary Sampling Units (PSUs) were obtained by the systematic random selection of 5 out of the 11 constituencies that were listed alphabetically and were assigned serial numbers. The five parliamentary constituencies selected were: Al-Keisha; Al-Sabre; Al-Salmani-ElSharki; Bu Atni and Madinat Benghazi. Each of the eleven constituencies has three polling districts (Benghazi Municipal Election, 2012) [50,51]. In the second stage, Secondary Sampling Units (SSUs) were derived by a simple random sampling of one polling district from each of the five constituencies selected, PSUs, specifically: Al-Fuwayhat; Al-Kwayfiya; Raas Abayda; Laithi and Al-Hadaa’iq, which respectively pertaining to Al-Keisha; Al-Sabre; Al-Salmani-ElSharki; Bu-Atni and Madinat Benghazi. Finally, in the third stage, Tertiary Sampling Units (TSUs) were obtained by the systematic random sampling of potential participants. Sampling technique is showed in Table 1.

### 2.1. Participants and Procedure 

The participant eligibility criteria were: aged 20–65 years; resident in Benghazi for over ten years; speaks an Arabic language and; eligible to register to vote. Additional exclusion criteria were pregnant women; people unsteady on their feet; chair-bound; amputees; and those too frail or unable to stand upright.

The study used the services of the “Alfwehat Telecom and Technology Agency”, which is affiliated to the Libyan general postal services. The agency allocated two males and two females to achieve this task and to overcome any gender communication barriers. Questionnaires were administered by the researchers with the participants. Meeting locations were varied, ranging from the participants’ homes to a clinic, other public places or, a meeting at a mutually agreeable place if requested by the participants. Most females opted to meet at the clinics in the regions where they live.

Given that Libyans have a conservative culture and religion [52] and that a number of anthropometric measurements were required from females, the study sought and obtained consent from the regional health sector in Benghazi to allocate male and female nurses to each of the polling districts. Female nurses were allocated to take the anthropometric measurements of female participants, aligned with cultural norms. Weight measurement was included using the [53], standard protocol for the use of the Tanita Segmental Body Composition Monitor. This helped to ensure the validity of the anthropometric measurements were taken, as well as facilitating to minimise religious and cultural obstacles, and maximising recruitment rates.

### 2.2. Ethical Considerations

Ethics approvals were granted from the following four relevant bodies: Institute of Health Research Ethics Committee (IHREC) at the University of Bedfordshire, UK, signed and dated on 28 January 2014, Omar Al-Mukhtar University, Bayda, Libya, signed and dated on 29 January 2014, and the Regional Health Ministry in Benghazi, Libya signed and dated on 30 January 2014. The research participants were fully informed about the nature and purpose of the study through a participant information document. Written signed and dated informed consent form was obtained from all research participants.

### 2.3. Measures

#### 2.3.1. Instrumentation for Taking Anthropometric Measurements

This study used a portable stadiometer to measure height (cm) to the nearest 0.1 cm. The Tanita BC-601 Segmental Body Composition Monitor was used to identify weight to the nearest 0.1 kg, BMI (kg/m^2^), Percent Body Fat nearest to 0.1% and Visceral Fat Level. 

#### 2.3.2. Sociodemographic Characteristics

The Arabic translated version of the WHO STEPS Instrument for Noncommunicable Disease Risk Factor Surveillance (only Step 1: Information on socio-demographic variables), was used to assess participants’ socio-demographic (e.g., age, gender, ethnicity, and religion) and socio-economic status. The latter was measured from questions pertaining to educational level, employment status, and income. Apart from Step 1 Demographic Information of WHO STEPS Instrument, this study disregarded the other scales of the same questionnaire namely, behavioural NCD risk factors, especially, the sections of alcohol intake, smoking, diet, physical activities and sedentary behaviour.

#### 2.3.3. Neighbourhood Environmental Assessment

The Physical Activity Neighbourhood Environment Scale (PANES) was adopted and used to investigate and assess the participant’s perceptions of neighbourhood environmental factors. PANES comprises 17-item that measure attributes of the built neighbourhood and social environments. Apart from using the PANES, this study did not use any further tool to collect data related to physical activities. To boost validity and reliability of this instruments use within the Libyan culture, we employed the following steps: (1) the forward-backward-forward translation technique through three professional bilingual Libyan certified translators and reviewed by an expert committee meeting; (2) Pre-test interviews with 10 Libyan adults were conducted; (3) The expert committee evaluated the final Arabic translated version, concluding the questionnaire to be straightforward, understandable, questions being in a regular sequence, and relevant to Libyans’ daily lives and; (4) using Pearson’s correlation coefficient to test for correlations between the score for each item of the questionnaire and the total score (r = 0.337, *p* < 0.001). The pre-test data were also tested for internal consistency using Cronbach’s alpha (α = 0.742).

#### 2.3.4. Statistical Analysis

The Statistical Package for Social Sciences (SPSS, version 22.0; SPSS Inc., Chicago, IL, USA) was used for data entry and analysis. The BMI pattern: BMI was coded into two categories as follows: not obese (<25.00) and obese (≥25.00), in order to perform a binary logistic regression analysis, which was performed to examine the relationship between the outcome variable (BMI) and, and neighbourhood environment attributes [49].

The income was categorized into six groupings based on Libyan Dinar/month (Libyan Dinar = ½ Pound). Income was recoded into three categories (low-income, moderate-income, and high income) for achieving the test of binary regression analysis. Using three categories has been used in other research studies [54,55].

Neighbourhood environment attributes aligned with previous studies in Japan by Inoue et al. (2009) [56], Ishii et al. (2010) [57] and Liao et al. (2011) [58], and another study conducted in Najera by Oyeyemi et al. (2012) [49], these studies used the following procedure to code and analysed the PANES instrument. The 17 environmental variables were converted into binary items in order to perform binary logistic regression analysis: Low residential density was coded as detached single-family housing. High residential density was coded as the other four housing types. A five-level Likert scale was used: Agree (strongly agree and agree), while Disagree (Neither, strongly disagree, disagree). 

A series of descriptive analyses were conducted to assess participants’ socio-demographic data and socio-economic status (SES), as well as the characteristics of participants with respect to the perception of environmental factors of the environmental neighbourhood. Logistic regression analyses were performed to examine the association between the neighbourhood environment attributes and obesity in Libyan adults and analysed according to the gender variance after adjusting for socio-demographic factors. In addition, logistic regression analysis was used to identify which, if any, neighbourhood environment attributes independently predicted obesity.

## 3. Results

The response rate achieved in this study was 78%, which was slightly higher than was expected. A total of 401 Libyan adults participated, 253 of whom were female (63.1%) and 148 were male (36.9%). The majority of the participants were married (67.1%), 84.6% were Arabic, 10.7% were Berbers ‘Imazighen’, and 4.7% were Toubou. 51.4% of the participants were with higher levels of educational attainment; 77.6% were employed, and 26% and 51% of the participants reported that they earned a low income and high income respectively. The mean BMI was 29.52 (±6.19) kg/m^2^, the mean visceral fat was 10.42 (±4.1), and the mean Body Fat Percentage was 31.57 (±9.42%). See Table 2 for a full breakdown of this data.

308 participants (76.8%) resided in high residential density zones. Obese people, who represented 84.5% (*n* = 256) of the sample, comprised the highest number of participants reported living in high-density areas. As can be seen in Table 3, 302 participants (75.5%) perceived crimes to be committed at night and reported feeling unsafe to walk in their neighbourhood. 287 (71.6%) perceived crimes to be committed during the day and reported feeling unsafe to walk in their neighbourhood. Obese people represented the highest percentage of participants who reported that crimes are occurring in their neighbourhood at night (*n* = 255; 84.2%), with 244 (80.5%) perceiving crimes to be occurring in their neighbourhood during the day.

A significant positive association between BMI and 6 of the 17 neighbourhood environment factors was identified, namely: residential density zones (Exp (B) = 3.33; 95% CI: 1.41–7.87), street connectivity (Exp (B) = 5.13; 95% CI = 2.25–11.70), unsafe environment and committing crimes at night (Exp (B) = 3.90; CI: 1.56–9.78), unsafe environment and committing crimes during the day (Exp (B) = 3.88; 95% CI: 1.52–9.89); (OR = 0.38; 95% CI: 0.15–0.96) and ‘aesthetics’ or the presence of beautiful things (Exp (B) = 0.08; 95% CI: 0.02–0.28). Street connectivity was the strongest predictors of outcomes compared to other factors in this model, linked to increased risk of obesity.

Examining the data of the male perceptions, there were positive associations between 6 of the 17 explanatory factors and developing BMI, including: access to public transport (Exp (B) = 0.02; 95% CI: 0.001–0.56); access to recreational facilities (Exp (B) = 0.03; 95% CI: 0.001–0.913); unsafe environment and committing crimes at night (OR = 27.76; 95% CI: 1.37–562.20); ‘aesthetics’ or the presence of beautiful things (Exp (B) = 0.001; 95% CI: 0.000–0.15); street connectivity (Exp (B) = 44.29; 95% CI: 2.69–729.63); and unsafe environment and committing crimes during the day (Exp (B) = 204.94; 95% CI: 3.15–13,336.90). For female Libyans, there were associations between 4 out of the 17 explanatory factors and developing BMI, specifically: residential density zones (Exp (B) = 4.77; 95% CI: 1.44–15.82); unsafe environment and committing crimes at night (Exp (B) = 5.90; 95% CI: 1.54–22.51); ‘aesthetics’ or the presence of beautiful things (Exp (B) = 0.01; 95% CI: 0.00–0.24); and street connectivity (Exp (B) = 5.75; 95% CI: 1.73–19.08). Street connectivity and the presence of beautiful places in females were the strongest predictors of outcomes compared to other factors in this model linked to increased risk of obesity (see Table 4).

## 4. Discussion

This study reveals a significant association between obesity in Libyan adults and 6 of the 17 neighbourhood environment factors. The present section briefly discusses and interprets the findings of each built neighbourhood environment factor separately.

### 4.1. Residential Density

Numerous factors could explain the significant positive association between ‘residential density zones’ and obesity found in women but not men. One possibility is that people who are living in low-density areas, such as in sprawling suburbs, tend to be car-dependent communities whereas those living in high-density areas such as central urban areas may have less reliance on cars [59,60]. Since driving a car is a relatively sedentary activity, those who rely on cars are more likely to have a higher BMI [27]. Another possibility is that the increasing residential density in Benghazi is leading to an overcrowding of open spaces, parks, public spaces, and pedestrian amenities, leaving little space for the residents to walk or undertake the outdoor physical activity. 

### 4.2. Unsafe Environment and Committing Crimes at Night or During the Day

The findings of this study reveal that the BMI of Libyan adults is significantly associated with ‘an unsafe environment and low perceived crime safety at night’ in Libyan adults in both genders, while with ‘an unsafe environment and low perceived crime safety during the day’ was significantly associated with BMI in men only. Most of the respondents in this study reported that they perceived the environment to be unsafe and perceived crime safety to be poor at night (71.6%) and during the day (75.5%). It may be inferred that they perceive the current situation in Benghazi to be very dangerous in all aspects, with some districts in Benghazi being evacuated by residents due to the fighting between the Libyan army and the radical Islamic militias. These factors may impede Libyans from undertaking physical activity in their daily lives. 

### 4.3. Access to Public Transport

Despite some 68% of participants both men and women in this study reporting that they are satisfied with access to public transport, many prefer to use their own vehicles. The findings diverged from those of literature in that ‘access to public transport’ was positively associated with obesity in men but not in women. A possible explanation is that Libyan men who reported higher use of or greater access to public transport had only a short distance to walk or to engage in physical activity, with the implication that they failed to reach the recommended level of physical activity [61]. Another possible explanation is that the destruction of infrastructure due to clashing armed groups contributes to Libyans’ reluctance to walk to use public transport. As vehicle users tend to be less physically active than those who use public transport, they may accordingly have a higher BMI. The final possible explanation for no association between access to public transport and obesity in Libyan women is that Libyan women encounter cultural and religious barriers which restrict them from utilising public transport, despite the provision of public transport in Benghazi. 

### 4.4. Pedestrian Infrastructure 

This study found that BMI of Libyan adults is not associated with pedestrian infrastructure in either gender. Although obese Libyan adults, who constituted the majority of participants in this study (63.8%), reported that their neighbourhoods have good pavements, and a similar proportion of participants reported that pavements were maintained (54.0%) and repaired (53.6%), the BMI of Libyan adults was not associated with pedestrian infrastructure (‘presence of pavements’, ‘maintenance of pavements’). One possible explanation is that consistent fighting between militias is creating unsafe neighbourhoods, such that Libyans are remaining indoors rather than going outside and risking injury or harm from militias. A second possibility is that infrastructure has been destroyed during this clashing between armed groups, making it difficult for Libyans to find suitable areas in which to practice physical activities, leading to an increase in their weight. 

### 4.5. Cycling Infrastructure 

A potential explanation for the lack of relationship between cycling infrastructure and obesity is the lack of car-free zones. These restrictions on motor-vehicle use in cities help to promote cycling and pedestrians. Thus, despite the existence of cycling lanes, the absence of such traffic regulations in Libya means that Libyans nevertheless tend to avoid cycling due to traffic hazards, and this lack of cycling might influence BMI. Women, in particular, prefer biking in low-traffic streets and feel more relaxed riding on off-street bike paths rather than on streets [62]. However, Libyan culture or Islamic laws restrict Libyan women from cycling. These constraints on their daily-life activities may contribute to an increase in obesity. In sum, although cycling infrastructure exists in Benghazi, Libyans are reluctant to engage in physical activities. 

### 4.6. Access to Recreational Facilities

Although this study reveals that the majority of Libyans (72.1%) perceive recreational facilities to be accessible, a positive association was found between access to recreational facilities and obesity in men, but not in women. One possible reason is that most find such facilities unaffordable and contrary to Islamic laws and cultural norms which require gender segregation. In addition, women face restrictions against engaging in physical activity, as some may interpret that Islam obligates women to wear a Scarf and veil; and cultural barriers can include clothing restrictions for women such that they cannot be seen in public wearing short sleeves. A final explanation is that most of these recreational facilities are deserted due to the conflict between outlaw militias, which the public is motivated to avoid. 

### 4.7. Traffic Safety

This study reveals that the BMI of Libyan adults is not associated with the traffic safety in either gender. Consensus exists among researchers that improving traffic safety through traffic-calming measures, such as incorporating auto-free zones throughout the city, may encourage and support the public to engage in physical activities, thereby helping to reduce the obesity risk [6,59]. The posited explanation for the lack of association between traffic safety measures and BMI in this study is the ongoing fighting among militias in Benghazi, which may discourage Libyans from practising their daily-life activities, thereby contributing to an increase in obesity rates. Another possible explanation is the ineffectiveness of the officials in preventing criminals from taking advantage of the country’s current circumstances by breaching the laws and entering auto-free zones, rendering these areas risky and unsuitable, for the public to practice daily physical activities.

### 4.8. Pedestrian Safety 

This study reveals that the BMI of Libyan adults is not associated with pedestrian safety in either gender. Improving pedestrian safety will inevitably improve walkability, which encourages physical activity and reduces the risk of obesity [6]. Despite some 67% of participants reporting that they perceive their neighbourhoods to have a high level of pedestrian safety, this study found no association between pedestrian safety and obesity. A possible explanation is that the conflicts between militias are resulting in unsafe neighbourhoods, with increased crime rates and traffic accidents. The pedestrian vulnerability is further exacerbated by Libyan traffic laws being inadequately enforced [63,64,65]. Another explanation the clashing between militias has damaged the pedestrian infrastructure, which encourages the public to stay at home rather than engaging in physical activity.

### 4.9. Neighbourhood Aesthetics

Surprisingly, this study found that the presence neighbourhood aesthetics is associated with a higher BMI in both genders. Neighbourhood aesthetics, such as the presence of street trees, is frequently shown to be important for physical activity through promoting physical activities [13]. The presence of street trees in Benghazi should provide a welcoming shade in the hot weather and should, in theory, create pleasant and safer routes for pedestrians and cyclists. One possible interpretation of our finding is that the unsafe neighbourhood environment created by the current conflicts constrains the public from enjoying these green spaces.

### 4.10. Vehicle Ownership

Another surprising finding of this study was the lack of an association between motor-vehicle ownership and obesity. Numerous studies have suggested that relying on vehicles for transport contributes to obesity. A possible explanation is that, despite most of the study participants (70.6%) reporting that their household owned multiple vehicles, the majority of Libyan adults may have been unwilling to drive their cars due to the socio-political instability and insecurity in Benghazi. There are also erratic and insufficient fuel supplies for automobiles in existing gas stations, as well as deterioration of the built environment in Benghazi due to intensive fighting between multiple militias, which resulted in reducing the number of vehicles on roads. Libyan women may have especially low vehicle use given they may feel inclined, for cultural or tribal reasons, on to rely upon male relatives to drive them. 

### 4.11. Limitations of the Present Study 

A possible weakness of the fieldwork environment was the unpredictable and escalating security risk due to the proliferation of Islamic State (ISIS) and other rebel militias fighting against the Libyan Armed Forces. Arguably, the broad eligibility criteria for the research participants included in this study adversely affected the response rate in that several groups were excluded due to practical reasons, for example, using a portable Tanita BC-601 Segmental Body Composition Monitor is impractical to provide physical anthropometric readings for certain groups of people include pregnant women; amputees; those unsteady on their feet, too frail or unable to stand upright, in fact, obtained, it may vary from the standard BMI cut-point values. Excluding these groups, as well as those prohibited from voting, whether for practical or legal reasons, it is arguably a limitation of this study as it did not take into account the perceptions of the entire population of Libyan. Another drawback, this study failed to collect further data concerning food consumption, alcohol consumption, physical activity, sedentary lifestyles and some habits such as smoking, which could be used as confounding variables for adjusting while performing the logistic regression analysis. A further limitation of this study is that Physical Activity and perceptions of the built environment characteristics were assessed using self-report questionnaires which revealed inconsistent results between neighbourhoods environment characteristics attributed and Physical Activity (PA). These results could vary when different data collection methods are used, such as objective measures to assess the geographical settings in which the respondents reside. To minimise this limitation, some researchers use a combination of subjective and objective methods [66].

## 5. Conclusions

The findings of this study concerning the relationship between neighbourhood environmental factors and obesity have revealed key areas for policy development in Libya. However, it is necessary to take into consideration how political instability within a country can influence day-to-day public behaviour due to potential security concerns. The study contributes useful findings to a limited knowledge-based relating to Arab countries who have similar built environments, cultural and religious norms. The study suggests that Libyan people are at risk of living in neighbourhoods with unsupportive environmental features of physical activity, which are likely to promote obesity of both genders. The findings of this study could inform Libyan health policies about interventions in the obesogenic environments that might slow the obesity epidemic and contain the public health crisis. This study suggests that further research is needed, within the Libyan context, to explore the impact of the neighbourhood environment attributes on contributing to increased obesity.

## Figures and Tables

**Table 1:** Sampling technique.

S.N.	The Randomly Selected Constituencies	The Randomly Selected Polling District	Number of Registered Voters in Each Polling District	% of the Calculated Sample Size in Each Polling District	Total Hypothesised Sample Size = 512	Total Actual Sample Size = 401
1.	Al-Keisha	Al-Fuwayhat	8012	13.8%	71	51
2.	Al-Sabre	Al-Kwayfiya	10,433	18%	92	75
3.	Al-Salmani-ElSharki	Raas Abayda	8480	14.7%	75	54
4.	Bu Atni	Laithi	11,858	20.5%	105	86
5.	Madinat Benghazi	Al-Hadaa’iq	19,102	33%	169	135
Total			57,885	100%	512	401

**Table 2:** Socio-demographic characteristics and anthropometric measurement vary by gender.

Demographic and Socio-Economic Characteristics	Male (M) N (%)	Female (F) N (%)	Total N (%)
Gender	148 (37)	253 (63)	401 (100)
Age			
20–29	27 (18)	51 (20)	78 (19)
30–39	27 (18)	56 (22)	83 (21)
40–49	37 (25)	78 (31)	115 (29)
50–59	18 (12)	32 (13)	50 (12)
60–65	39 (26)	36 (14)	75 (19)
Marital Status			
Single (Unmarried)	47 (31.8)	85 (33.6)	132 (32.9)
Married ^1^	101 (68.2)	168 (66.4)	269 (67.1)
Racial group			
Arabic	129 (87.2)	210 (83)	339 (84.6)
Berbers ‘Imazighen’	15 (10.1)	28 (11.1)	43 (10.7)
Toubou	4 (2.7)	15 (5.9)	19 (4.7)
Level of education			
Low educational level *	32 (21.6)	45 (17.8)	77 (19.2)
Moderate educational level **	41 (27.7)	77 (30.4)	118 (29.4)
High educational level ***	75 (50.7)	131 (51.8)	206 (51.4)
Occupation			
Employed groups ^2^	120 (81.1)	191 (75.5)	311 (77.6)
Unemployed groups ^3^	28 (18.9)	62 (24.5)	90 (22.4)
Monthly Income: “(LYD)” *			
Low income <999	46 (31)	57 (23)	103 (26)
1000–1999	8 (5)	17 (7)	25 (6)
Moderate income 2000–2999	16 (11)	54 (21)	70 (18)
High income: 3000–3999	35 (24)	60 (24)	95 (24)
4000–4999	25 (17)	53 (21)	78 (19)
≥5000	18 (12)	12 (5)	30 (7)
Anthropometric measurements	Male Mean (±SD)	Female Mean (±SD)	Participants Mean (±SD)
BMI values (kg/m^2^)	28.50 (±5.40)	30.12 (±6.54)	29.52 (±6.19)
Visceral Fat Rating (1–12)	10.04 (±3.9)	10.65 (±4.2)	10.42 (±4.1)
Body fat %	27.01 (±7.31)	34.24 (±9.51)	31.57 (±9.42)

Married ^1^: “Being married, divorced separated & widowed.” Low educational level *: “No formal schooling; Less than a primary school; Primary school completed.” Moderate educational level **: “Secondary school completed; High school completed.” High educational level ***: “College/university completed; Post graduate degree.” Employed groups ^2^: “Government employee; non-government employee; Self-employed; non-paid & student.” Unemployed groups ^3^: “Housework; retired; unemployed (able to work); and unemployed (unable to work). (Libyan Dinar (LYD) * = 1/2 Pound under current exchange rate).

**Table 3:** Association between neighbourhood environment factors and BMI using binary logistic regression when sociodemographic and SES factors were adjusted *.

Neighbourhood Environment Variables	The Response Options	Responses of Non-Obese Participants BMI < 25.00 N (%)	Responses of Obese Participants BMI ≥ 25.00 N (%)	Total Participants N (%)	Sig.	Exp (B)	95% CI for Exp (B)	
							Lower	Upper
1. Residential density.	Low	46 (47)	47 (15.5)	93 (23.2)	0.006	3.33	1.41	7.87
	High	52 (53)	256 (84.5)	308 (76.8)				
2. Access to commercial places.	Agree	20 (20.4)	102 (33.7)	122 (30.4)	0.269	0.55	0.19	1.60
	Disagree	78 (79.6)	201 (66.3)	279 (69.6)				
3. Access to public transport.	Agree	17 (17.3)	112 (37)	129 (32.2)	0.040	0.38	0.15	0.96
	Disagree	81 (82.7)	191 (63)	272 (67.8)				
4. Presence of pavements.	Agree	35 (35.7)	86 (28.4)	121 (30.2)	0.599	1.37	0.42	4.45
	Disagree	63 (64.3)	217 (71.6)	280 (69.8)				
5. Presence of cycle lanes.	Agree	40 (40.8)	198 (65.3)	238 (59.4)	0.118	0.40	0.13	1.26
	Disagree	58 (59.2)	105 (34.7)	163 (40.6)				
6. Access to recreational facilities.	Agree	20 (20.4)	92 (30.4)	112 (27.9)	0.453	0.69	0.26	1.81
	Disagree	78 (79.6)	211 (69.6)	289 (72.1)				
7. Crime/safety at night.	Agree	50 (51)	48 (15.8)	98 (24.4)	0.004	3.90	1.56	9.78
	Disagree	48 (49)	255 (84.2)	303 (75.6)				
8. Traffic safety.	Agree	52 (53)	96 (31.7)	148 (36.9)	0.211	1.71	0.74	3.93
	Disagree	46 (47)	207 (68.3)	253 (63.1)				
9. See people as being active.	Agree	40 (40.8)	92 (30.4)	132 (32.9)	0.894	0.95	0.42	2.15
	Disagree	58 (59.2)	211 (69.6)	269 (67.1)				
10. Presence of beautiful things ‘Aesthetics’.	Agree	8 (7)	123 (41)	131 (32.7)	0.000	0.08	0.02	0.28
	Disagree	91 (93)	179 (59)	270 (67.3)				
11. Household vehicle ownership.	Agree	40 (40.8)	78 (25.7)	118 (29.4)	0.869	0.93	0.40	2.19
	Disagree	58 (59.2)	225 (74.3)	283 (70.6)				
12. Connectivity of streets.	Agree	55 (56)	82 (27)	137 (34.2)	0.000	5.13	2.25	11.70
	Disagree	43 (44)	221 (73)	264 (65.8)				
13. Maintenance of pavements.	Agree	31 (31.6)	88 (29)	119 (29.7)	0.576	1.43	0.41	4.98
	Disagree	67 (68.4)	215 (71)	282 (70.3)				
14. Maintenance of cycle lanes.	Agree	36 (36.7)	169 (55.7)	205 (51.1)	0.742	0.82	0.25	2.66
	Disagree	62 (63.3)	134 (44.3)	196 (48.9)				
15. Traffic safety for cyclists.	Agree	51 (52)	87 (28.7)	138 (34.4)	0.658	0.82	0.34	1.98
	Disagree	47 (48)	216 (71.3)	263 (65.6)				
16. Crime/safety during the day.	Agree	55 (56.1)	59 (19.5)	114 (28.4)	0.004	3.88	1.52	9.89
	Disagree	43 (43.9)	244 (80.5)	287 (71.6)				
17. Public places and walkable destinations.	Agree	23 (23.5)	119 (39.3)	142 (35.4)	0.856	0.91	0.32	2.61
	Disagree	75 (76.5)	184 (60.7)	259 (64.6)				

OR: odds ratio; CI: confidence interval. The OR was calculated for all variables with 95% confidence intervals. * Adjusted for, gender, age, marital status, level of education, occupation and income.

**Table 4:** Association between neighbourhood environment factors and BMI using binary logistic regression across gender-related variances when sociodemographic and SES factors were adjusted *.

Neighbourhood Environment Variables	Male				Female			
	Sig. *p*-Value	Odds Ratio (OR)	95% CI for Exp (B)		Sig. *p*-Value	Odds Ratio (OR)	95% CI for Exp (B)	
			Lower	Upper			Lower	Upper
1. Residential density.	0.092	9.13	0.70	120.02	0.011	4.77	1.44	15.82
2. Access to commercial places.	0.295	5.62	0.22	141.87	0.110	0.28	0.06	1.34
3. Access to public transport.	0.022	0.02	0.001	0.56	0.317	0.54	0.16	1.80
4. Presence of pavements.	0.118	43.76	0.38	4997.11	0.869	0.86	0.15	5.01
5. Presence of cycle lanes.	0.139	0.04	0.00	2.82	0.445	0.54	0.11	2.62
6. Access to recreational facilities.	0.044	0.03	0.001	0.913	0.935	0.95	0.25	3.66
7. Crime/safety at night.	0.030	27.76	1.37	562.20	0.009	5.90	1.54	22.51
8. Traffic safety.	0.948	0.93	0.09	9.33	0.494	1.57	0.43	5.77
9. See people as being active.	0.521	2.28	0.184	28.29	0.151	0.40	0.11	1.40
10. Presence of beautiful things ‘Aesthetics’.	0.007	0.001	0.000	0.12	0.003	0.01	0.00	0.24
11. Household vehicle ownership.	0.538	0.47	0.042	5.21	0.580	0.72	0.22	2.34
12. Connectivity of streets.	0.008	44.29	2.69	729.63	0.004	5.75	1.73	19.08
13. Maintenance of pavements.	0.383	5.249	0.127	216.23	0.677	0.68	0.12	4.24
14. Maintenance of cycle lanes.	0.304	0.13	0.003	6.48	0.455	0.54	0.10	2.75
15. Traffic safety for cyclists.	0.056	0.02	0.001	1.10	0.585	1.43	0.40	5.20
16. Crime/safety during the day.	0.012	204.94	3.15	13,336.90	0.095	2.97	0.83	10.64
17. Public places and walkable destinations.	0.985	0.97	0.048	19.86	0.513	0.61	0.14	2.72

OR: odds ratio; CI: confidence interval. The OR was calculated for all variables with 95% confidence intervals. * Adjusted for, gender, age, duration of residence in Benghazi, marital status, level of education, occupation and income.

## Declaration

The data in this paper largely from a thesis submitted by Hamdi Abdulla A. Lemamsha in partial fulfillment of the requirements for the PhD degree in Exploring the risk and protective factors associated with obesity amongst Libyan adults, University of Bedfordshire, 2016.

## Abbreviations

BMI	Body Mass Index
HNEC	High National Election Commission
MCS	Multistage Cluster Sample
NE	Neighbourhood Environment
BE	Built Environment
BEEs	Built Environment Experts
PANES	Physical Activity Neighbourhoods Environment Survey
SEM	Socio-Ecological Model
